# m5CStack: An integrated framework for m5C site prediction using multi-feature stacking

**DOI:** 10.1016/j.csbj.2025.05.004

**Published:** 2025-05-12

**Authors:** Xuxin He, Jiahui Guan, Peilin Xie, Zhihao Zhao, Qianchen Liu, Lantian Yao, Ying-Chih Chiang

**Affiliations:** a Kobilka Institute of Innovative Drug Discovery, School of Medicine, The Chinese University of Hong Kong, Shenzhen, 2001 Longxiang Road, 518172, Shenzhen, China; b School of Medicine, The Chinese University of Hong Kong, Shenzhen, 2001 Longxiang Road, 518172, Shenzhen, China; c School of Science and Engineering, The Chinese University of Hong Kong, Shenzhen, 2001 Longxiang Road, 518172, Shenzhen, China

**Keywords:** 5-methylcytosine, RNA modification, Machine learning, Ensemble learning, Stacking architecture

## Abstract

RNA 5-methylcytosine (m5C) modification sites are essential for understanding the regulation of RNA functions in various biological processes. However, the vast amount of sequence data generated by modern genomics poses significant challenges for traditional identification methods, which often struggle to meet high-throughput demands. Consequently, computational tools have become indispensable for predicting m5C sites. In this study, we present m5CStack, an advanced ensemble learning framework designed to predict m5C modification sites with high accuracy. m5CStack integrates multiple feature encoding techniques and machine learning models through a stacking architecture to enhance the robustness and reliability of predictions. We evaluate the framework on RNA datasets derived from multiple species, including *Homo sapiens* (human), *Mus musculus* (mouse), *Drosophila melanogaster* (drosophila), and *Danio rerio* (danio). Experimental results demonstrate that m5CStack significantly outperforms previous prediction methods across a range of metrics, including accuracy, sensitivity, and specificity. Furthermore, SHAP-based feature significance analysis reveals the key contribution of specific features, further improving the interpretability of the model. To improve accessibility, a user-friendly web interface is developed, allowing users to input RNA sequences or upload files for prediction, with results displayed in an intuitive format alongside confidence scores. Overall, this study highlights the potential of m5CStack as a powerful tool for RNA modification profiling, offering new insights into the epigenetic regulation of RNA across species.

## Graphical abstract

**Figure:**
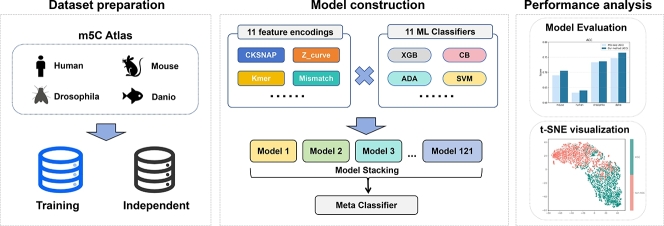

## 1 Introduction

In recent years, studies of epigenetic modifications have demonstrated their crucial involvement in regulating diverse biological processes, spanning from gene expression control to pathological development. These molecular alterations, including RNA methylation and histone modifications, exert profound influences on cellular functions through sophisticated regulatory mechanisms, even in the absence of DNA sequence changes [1], [2], [3]. Among these modifications, 5-methylcytosine (m5C) has emerged as one of the most abundant and well-studied marks in mammalian RNA [4], [5]. Characterized by the addition of a methyl group to the carbon-5 position of cytosine residues within RNA molecules, m5C plays a multifaceted role in modulating RNA metabolism, including translation efficiency, stability, ribosome biogenesis, and the export of mRNA from the nucleus [6], [7], [8]. Furthermore, m5C is crucial in regulating mRNA turnover, which is essential for maintaining cellular homeostasis [9]. The widespread presence of m5C across various RNA species has highlighted its fundamental role in maintaining normal cellular processes [10], [11], [12]. Importantly, accumulating evidences have firmly established connections between aberrant m5C levels and diverse pathological states, particularly cancer progression and neurodegenerative disorders [13], [14], [15], underscoring the importance of understanding its role in disease mechanisms. Given the extensive involvement of m5C modification in crucial RNA functional processes, this epigenetic mark has become a pivotal research subject for deciphering epigenetic regulatory networks and their health implications.

Despite the biological importance of m5C modifications, accurately and efficiently identifying m5C sites in RNA has remained a challenge due to the large amount of sequence data generated by modern genomics. Traditional experimental methods, such as bisulfite sequencing, transmission electron microscopy [16], [17], provide high-precision identification, but are often unsuitable for scaling to the high-throughput data produced in post-genomic studies [18]. To address these limitations, computational approaches, particularly machine learning (ML), have emerged as powerful tools for m5C site prediction [19], [20], [21]. ML models excel at processing large datasets and identifying complex patterns that might be difficult to detect with conventional methods. For example, Feng et al. [22] proposed an SVM-based model that utilizes pseudo k-tuple nucleotide composition (PseKNC) to predict m5C sites in RNA sequences. Building upon this concept, Qiu et al. developed iRNAm5C-PseDNC [23], which employed a similar encoding approach based on PseDNC. Subsequently, Zhang et al. [24] proposed M5C-HPCR, employed heuristic nucleotide properties and ensemble classification techniques to improve prediction accuracy. Furthermore, Sabooh et al. [25] combined SVM (support vector machines) with a novel encoding technique to further enhance m5C site prediction, while Li et al. [26] developed RNAm5Cfinder, an online tool that uses random forests to predict m5C sites across different tissues. Additionally, Abbas et al. [27] proposed m5C-pred, an XGBoost framework with feature selection for the prediction of m5C sites.

However, current ML approaches exemplified by iRNAm5C-PseDNC [23] and RNAm5CPred [28] are constrained by their dependence on limited training datasets and insufficient external validation. These limitations, including overfitting and reduced generalizability, become particularly evident when applying these models to broader biological contexts. While these frameworks have offered important mechanistic insights, their technical robustness remains inadequate for large-scale biomedical applications. To address these challenges, more advanced tools, such as m5C-Seq proposed by Abbas et al. [29], have been developed. By utilizing a large-scale, diverse dataset and incorporating different feature encoding techniques alongside ML classifiers, m5C-Seq improved the accuracy of m5C site prediction. However, it still encountered difficulties in capturing complex sequence patterns and ensuring robustness across diverse biological contexts. Moreover, this method has yet to offer a user-friendly web platform, restricting broader accessibility and direct application by other researchers.

To address the challenges in RNA modification profiling, we introduce m5CStack, an advanced ensemble learning framework for detecting m5C sites in RNA. m5CStack utilizes a large-scale, diverse dataset and employs 11 distinct feature encoding techniques to capture a comprehensive representation of RNA sequences. These features are processed through 11 ML classifiers, generating 121 baseline models. The predictions from these models are combined using a stacking approach, where a meta-classifier optimally combines the outputs for final prediction. This strategy enhances the model's ability to handle complex sequence patterns, thereby improving its accuracy, sensitivity, and robustness. By leveraging the diversity of 11 feature encodings and 11 classifiers, m5CStack captures a broad spectrum of sequence information, ensuring a more precise and comprehensive identification of m5C sites. Each classifier contributes a unique perspective, improving the model's robustness through error reduction. The stacking strategy further exploits the strengths of individual classifiers, resulting in more reliable predictions compared to models with fewer classifiers. Rigorous validation across multiple species demonstrates that m5CStack outperforms existing tools in terms of prediction accuracy and generalizability, providing a reliable and powerful approach for advancing the understanding of m5C modifications and their role in epigenetic regulation.

## 2 Materials and methods

### 2.1 Dataset preparation

In this study, we utilized the dataset collected by Abbas et al. [29] from the m5C-Atlas database [30], which includes four species: *Homo sapiens* (human), *Drosophila melanogaster* (drosophila), *Mus musculus* (mouse), and *Danio rerio* (danio). The m5C-Atlas database is a high-confidence collection of m5C sites derived from 351 bisulfite sequencing samples across 13 species, including human, mouse, zebrafish, and others. The raw data were obtained from the NCBI GEO database [31] and underwent quality control using Trim Galore [32]. After cleaning, the reads were mapped to reference genomes using meRanTK [33], and m5C sites were identified using meRanCall [33]. The false discovery rate was controlled at 0.01, with a minimum coverage threshold set at 30 reads.

Our dataset was constructed based on the above data collection methods, consisting of m5C-modified (positive) and non-modified (negative) RNA sequences from the four species, divided into training and independent validation sets. Each sequence is 41 bp in length, centered on a cytosine nucleotide, with 20 bp upstream and 20 bp downstream to capture the modification context. The training set was used for model development, while the independent validation set was used to evaluate the model's generalization capability. Table 1 provides details on the sample sizes and dataset partitioning for each species. This dataset laid the foundation for training m5CStack, enabling the model to capture the diversity of m5C sites and ensure reliable predictions across different biological contexts.

**Table 1:** Summary of m5C site datasets.

Category	Dataset	Positive	Negative	Total
Training data	*Homo sapiens*	16,979	16,979	33,958
	*Mus musculus*	131,086	131,086	262,172
	*Drosophila melanogaster*	6,020	6,020	12,040
	*Danio rerio*	9,628	9,628	19,256

Independent data	*Homo sapiens*	4,244	4,244	8,488
	*Mus musculus*	32,772	32,772	65,544
	*Drosophila melanogaster*	1,504	1,504	3,008
	*Danio rerio*	2,408	2,408	4,816

### 2.2 Overall framework of m5CStack

**Fig. 1:**
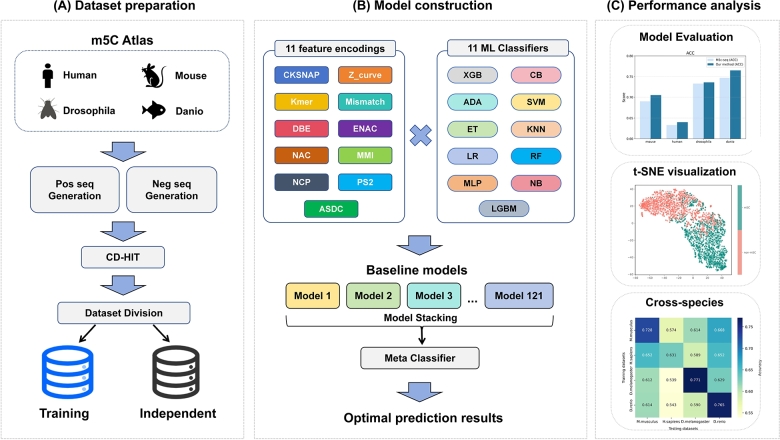
The workflow of the m5CStack, including: (A) Dataset preparation involves collecting RNA sequences from multiple species (human, mouse, drosophila, and danio) from the m5C-Atlas database, followed by the generation of positive and negative samples and dataset division into training and independent sets. (B) Model construction integrates 11 feature encoding techniques and 11 ML classifiers, with model stacking and a Random Forest meta-classifier used to enhance prediction accuracy. (C) Performance analysis incorporates various evaluation methods, including model evaluation metrics, t-SNE visualizations, and cross-species heatmaps, to comprehensively assess the model's accuracy and generalizability across different species.

The architecture of m5CStack is illustrated in Fig. 1. This framework is designed to address the challenges in RNA modification profiling by providing a reliable and accurate solution for predicting RNA 5-methylcytosine (m5C) sites. m5CStack employs an advanced ensemble learning strategy that integrates 11 distinct feature encoding techniques and 11 ML classifiers, resulting in 121 baseline models. These models are combined through a stacking architecture, where a Random Forest meta-classifier optimally aggregates their predictions to enhance overall performance.

By exploiting the diversity of feature codes and classifiers, m5CStack captures a wide range of sequence information, enabling robust pattern recognition and accurate identification of m5C sites. This approach not only improves prediction accuracy, but also ensures high sensitivity, specificity and generalizability across multiple species. Rigorous validation shows that m5CStack outperforms existing tools and serves as a robust and reliable solution for advancing the understanding of m5C modifications and their role in epigenetic regulation.

### 2.3 Feature encodings in m5CStack

In m5CStack, a comprehensive set of 11 feature encoding techniques is used to capture the intricate patterns of RNA sequences related to m5C modifications. These feature encodings include: CKSNAP, Z_curve, Kmer, Mismatch, DBE, ENAC, NAC, MMI, NCP, PS2, ASDC [34].

These encoding methods extract various sequence properties and statistical patterns, ensuring that the model effectively captures both local and global features of RNA sequences. By utilizing a diverse range of feature representations, m5CStack enhances its ability to model RNA sequences comprehensively, thereby improving performance and generalization across different species. Detailed descriptions of these feature encoding algorithms are provided in the supplementary materials.

### 2.4 Classifiers in m5CStack

To process the extracted features, m5CStack employs an ensemble of 11 ML classifiers, each contributing unique perspectives to the final prediction. These classifiers include: XGBoost (XGB), CatBoost (CB), AdaBoost (ADA), Support Vector Machine (SVM), ExtraTrees (ET), K-Nearest Neighbors (KNN), Logistic Regression (LR), Random Forest (RF), Multilayer Perceptron (MLP), Naive Bayes (NB), LightGBM (LGBM) [35].

Each classifier is trained independently on the RNA sequence features, assessing their significance and generating individual predictions. By combining the outputs of these models, m5CStack leverages the strengths of each, ensuring robust and reliable performance. The diverse ensemble enhances the model's capability to handle heterogeneous data types, thereby improving overall predictive accuracy. The hyperparameter settings of these classifiers are provided in Table S1.

### 2.5 Stacking ensemble strategy

Stacking architecture has demonstrated its effectiveness in enhancing the predictive accuracy of computational and bioinformatics tools, including antiviral peptides prediction [36], RNA-protein interaction prediction [37], and drug-induced liver injury risk prediction [38]. In m5CStack, we utilized a stacking architecture to integrate the predictions from multiple classifiers, aiming to improve the robustness and accuracy of m5C site prediction.

The stacking strategy consists of two levels: base classifiers and a meta-classifier. In the first level, multiple base classifiers (e.g., XGBoost, Logistic Regression, and Support Vector Machines) are trained independently on the same input data. Each base classifier generates its own set of predictions based on the input features. These predictions are then used as input features for the second level, where a meta-classifier (e.g., Random Forest or another ensemble model) is trained to combine the outputs of the base classifiers. The meta-classifier learns to weigh the predictions from the base classifiers optimally, thereby producing a final prediction that leverages the strengths of each individual model.

This ensemble strategy enhances model performance by capturing diverse data patterns that individual models may overlook [39]. By combining the predictions of multiple classifiers, the stacking approach reduces the risk of overfitting and improves generalization to unseen data. In our implementation, we carefully selected base classifiers, ensuring that the meta-classifier can effectively integrate their predictions. The final model, m5CStack, thus benefits from the collective intelligence of multiple classifiers, leading to more accurate and reliable m5C site predictions.

### 2.6 Voting ensemble strategy

In addition to the stacking ensemble approach, we implemented a voting ensemble strategy as an alternative model combination method. The voting ensemble operates on the principle of collective decision-making from multiple base classifiers. This method involves training several heterogeneous classifiers independently, including XGBoost, Random Forest, and Support Vector Machines, which possess distinct algorithmic characteristics and inductive biases.

The voting mechanism functions differently for classification versus regression tasks. For classification, it employs majority voting where the class prediction receiving the most votes from the base classifiers becomes the final output. In regression tasks, it utilizes averaging to combine the continuous outputs from all base models. The voting ensemble can be implemented in two variants: hard voting uses direct class labels from the classifiers, while soft voting operates on predicted class probabilities, often yielding more refined predictions by considering the confidence levels of each classifier's output.

The fundamental advantage of voting ensembles lies in their ability to reduce variance through model averaging while mitigating overfitting by leveraging model diversity. This approach capitalizes on the “wisdom of crowds” principle, where the collective decision of multiple models typically outperforms individual classifiers, especially when the base models exhibit low correlation in their errors. The computational efficiency of voting ensembles is noteworthy as they eliminate the need for training a secondary meta-model while still benefiting from ensemble effects.

### 2.7 t-SNE for dimensionality reduction and visualization

For feature space analysis and visualization, we employed t-Distributed Stochastic Neighbor Embedding (t-SNE) [40], a nonlinear dimensionality reduction technique. t-SNE operates by modeling pairwise similarities in both high-dimensional and low-dimensional spaces. The algorithm begins by computing probability distributions that represent pairwise similarities in the original high-dimensional space, then constructs an equivalent probability distribution in the target low-dimensional space (typically 2D or 3D), and finally minimizes the Kullback-Leibler divergence between these two distributions.

Compared to linear dimensionality reduction techniques like Principal Component Analysis (PCA), t-SNE excels at revealing intricate manifold structures and preserving local neighborhood relationships in complex datasets. The computational complexity of t-SNE is mitigated through the Barnes-Hut approximation, which enables efficient processing of moderately-sized datasets. This makes t-SNE particularly valuable for exploratory data analysis in biological and biomedical applications where understanding cluster patterns in high-dimensional feature spaces is crucial.

### 2.8 SHAP for model interpretation

To gain deeper insights into the decision-making process of our model, we employed SHAP (Shapley Additive Explanations) [41] for model interpretation. SHAP is a game-theoretic approach that quantifies the contribution of each feature to a model's predictions. It is based on Shapley values, a concept from cooperative game theory, which fairly distributes the total gain (or loss) among all players (features) by considering all possible feature combinations.

For our m5C site prediction model, SHAP was used to analyze feature importance across different feature extraction methods. By computing SHAP values for each sample, we identified which features contributed most significantly to the model's classification decisions. This allowed us to not only improve the interpretability of our machine learning model but also derive biological insights into the distinguishing characteristics of m5C sites.

### 2.9 Evaluation metrics

In this study, we evaluated the model's performance using Accuracy, Sensitivity, Specificity, F1-score, and the Matthews Correlation Coefficient (MCC) as key metrics [42]. They are defined as follows:

(1) $$\text{Accuracy}=\frac{TP+TN}{TP+TN+FP+FN}$$

(2) $$\text{Sensitivity}=\frac{TP}{TP+FN}$$

(3) $$\text{Specificity}=\frac{TN}{TN+FP}$$

(4) $$F1-\text{score}=2\times \frac{TP}{2TP+FN+FP}$$

(5) $$MCC=\frac{TP\times TN-FP\times FN}{\sqrt{\left(TP+FP)(TP+FN)(TN+FP)(TN+FN\right)}}$$

These metrics are computed based on true positives (TP), true negatives (TN), false positives (FP), and false negatives (FN). Additionally, the Area Under the Curve (AUC) of the Receiver Operating Characteristic (ROC) curve was calculated, ranging from 0.5 to 1. Both ROC curves and AUC values provide a comprehensive evaluation of the model's predictive performance [43].

## 3 Results and discussion

### 3.1 Evaluation of model performance in comparison to existing methods

Table 2 presents the average results of 5-fold cross-validation across four species. The accuracy ranges from 0.640 in the human dataset to 0.765 in the danio dataset, with danio achieving the best overall performance. Sensitivity and specificity remain relatively balanced across all datasets, with values typically within a 0.63–0.77 range, ensuring that the model effectively identifies both positive and negative instances. The MCC values range from 0.279 to 0.530, reflecting a solid predictive power, particularly in the danio and drosophila datasets, where MCC exceeds 0.47. These results suggest that the model generalizes well and captures meaningful patterns in m5C site prediction.

**Table 2:** Performance evaluation based on the average results of 5-fold cross-validation on the training dataset.

Datasets	Accuracy	Sensitivity	Specificity	F1-score	MCC
*Mus musculus*	0.705	0.714	0.696	0.707	0.409
*Homo sapiens*	0.640	0.630	0.649	0.626	0.279
*Drosophila melanogaster*	0.736	0.738	0.735	0.736	0.472
*Danio rerio*	0.765	0.770	0.759	0.766	0.530

As shown in Table 3, the model maintains strong performance on the independent dataset, with accuracy values ranging from 0.631 to 0.773. Drosophila achieves the highest accuracy (0.773) and MCC (0.547), while danio also performs well with an MCC of 0.531. Sensitivity and specificity remain balanced across species, with specificity reaching 0.798 in the drosophila dataset, indicating strong predictive confidence. The F1-score range from 0.624 to 0.768, demonstrating the model's ability to consistently distinguish between m5C and non-m5C sites. These results confirm that the model generalizes effectively across different species and independent datasets.

**Table 3:** Performance evaluation on independent dataset.

Datasets	Accuracy	Sensitivity	Specificity	F1-score	MCC
*Mus musculus*	0.728	0.722	0.734	0.727	0.457
*Homo sapiens*	0.631	0.622	0.650	0.624	0.262
*Drosophila melanogaster*	0.773	0.749	0.798	0.768	0.547
*Danio rerio*	0.765	0.778	0.753	0.768	0.531

Table 4 highlights the superior performance of our model m5CStack on multiple evaluation metrics compared to existing models including iRNA-m5C [44], m5C-pred and m5C-seq. In terms of accuracy, sensitivity, specificity, F1-score, and MCC, m5CStack achieved the highest values in most of the metrics, demonstrating its reliability in m5C site prediction.

**Table 4:** Performance comparison on independent dataset. The best results are highlighted in bold.

Datasets	Methods	Accuracy	Sensitivity	Specificity	F1-score	MCC
*Mus musculus*	iRNA-m5C	0.505	**0.807**	0.502	0.023	0.052
	m5C-pred	0.620	0.618	0.622	0.619	0.239
	m5C-seq	0.707	0.713	0.701	0.702	0.413
	m5CStack	**0.728**	0.722	**0.734**	**0.727**	**0.457**

*Homo sapiens*	iRNA-m5C	0.482	0.481	0.482	0.481	0.030
	m5C-pred	0.526	0.232	**0.820**	0.329	0.065
	m5C-seq	0.618	**0.624**	0.612	0.607	0.236
	m5CStack	**0.631**	0.622	0.650	**0.624**	**0.262**

*Drosophila melanogaster*	m5C-seq	0.758	**0.761**	0.755	0.756	0.516
	m5CStack	**0.773**	0.749	**0.798**	**0.768**	**0.547**

*Danio rerio*	m5C-seq	0.743	0.729	**0.760**	0.751	0.487
	m5CStack	**0.765**	**0.778**	0.753	**0.768**	**0.531**

For mouse dataset, m5CStack achieves an accuracy of 0.728, outperforming m5C-seq (0.707) while also reaching the highest MCC (0.457). Similarly, in the human dataset, m5CStack surpasses both m5C-seq (0.618) and m5C-pred (0.526) with an accuracy of 0.631. In the drosophila and danio datasets, m5CStack continues to demonstrate strong predictive capabilities, achieving the highest accuracy (0.773 and 0.765, respectively). Some existing methods show imbalanced sensitivity and specificity, such as iRNA-m5C in the mouse dataset, which has a sensitivity of 0.807 but a specificity of only 0.502. In contrast, m5CStack maintains a well-balanced performance, ensuring both high sensitivity and specificity across different datasets.

Overall, m5CStack demonstrates superior performance in terms of accuracy, sensitivity, specificity, MCC, AUC, and F1-score across all species datasets, indicating that the proposed ensemble learning approach significantly improves RNA m5C site prediction compared to the existing methods. This enhancement in performance can be attributed to the combination of multiple feature encoding techniques, the use of diverse classifiers, and the integration of predictions through a meta-classifier, which collectively improve the robustness and generalizability of the model. In conclusion, m5CStack proves to be an effective method for RNA m5C site prediction, outperforming existing models like m5C-seq and providing more reliable results across multiple species. Bar charts illustrating the performance comparisons are shown in Figure S1 and Figure S2 of the supplementary materials.

### 3.2 Cost-benefit analysis of ensemble complexity

To evaluate whether the performance gains of integrating 121 models (11 classifiers × 11 feature encodings) justify the added complexity, we compared our full ensemble with a simplified approach where the 11 feature encodings were concatenated into a single vector and fed into the same 11 classifiers (reducing the ensemble to 11 models).

This simplified approach reduces computational complexity while maintaining the diversity of feature representations and classifiers. The experiment was conducted on the mouse dataset. And the results of the performance comparison between our method and the simplified method are summarized in Table S3.

As shown in Table S3, our original method significantly outperforms the simplified approach across all evaluation metrics. Specifically, our method achieves an accuracy of 0.728 compared to 0.693 for the simplified method, representing a 3.5% improvement. Similarly, the AUC score improves from 0.732 to 0.783, demonstrating a substantial gain in predictive performance. The F1-score and MCC also show notable improvements, further highlighting the superiority of our original approach.

To objectively compare computational costs, both the 121-model ensemble and simplified 11-model version were tested on the same reduced-scale dataset (a randomly selected 10% subset of the mouse data). Table S4 compares the training and testing times between these two methods. The original method integrates 121 models and requires approximately twice the training time (90.728 seconds versus 42.590 seconds) and testing time (10.702 seconds versus 4.580 seconds) compared to the simplified method that uses only 11 models. This represents a modest increase in computational time considering the significant expansion in model complexity. While the original method demands more computational resources, the enhanced performance in terms of accuracy, AUC and other key metrics as demonstrated in Table S3 fully justifies this additional cost. Such performance improvements are particularly valuable for important applications including disease biomarker discovery and functional RNA analysis. In these research areas, even minor enhancements in predictive capability can lead to meaningful biological discoveries and clinical applications.

In conclusion, the results demonstrate that the original 121-model ensemble, while more computationally intensive, provides significant performance advantages over the simplified approach. This justifies the added complexity, as our method delivers superior accuracy and robustness.

### 3.3 Performance analysis of different feature selection and base classifier choices

**Fig. 2:**
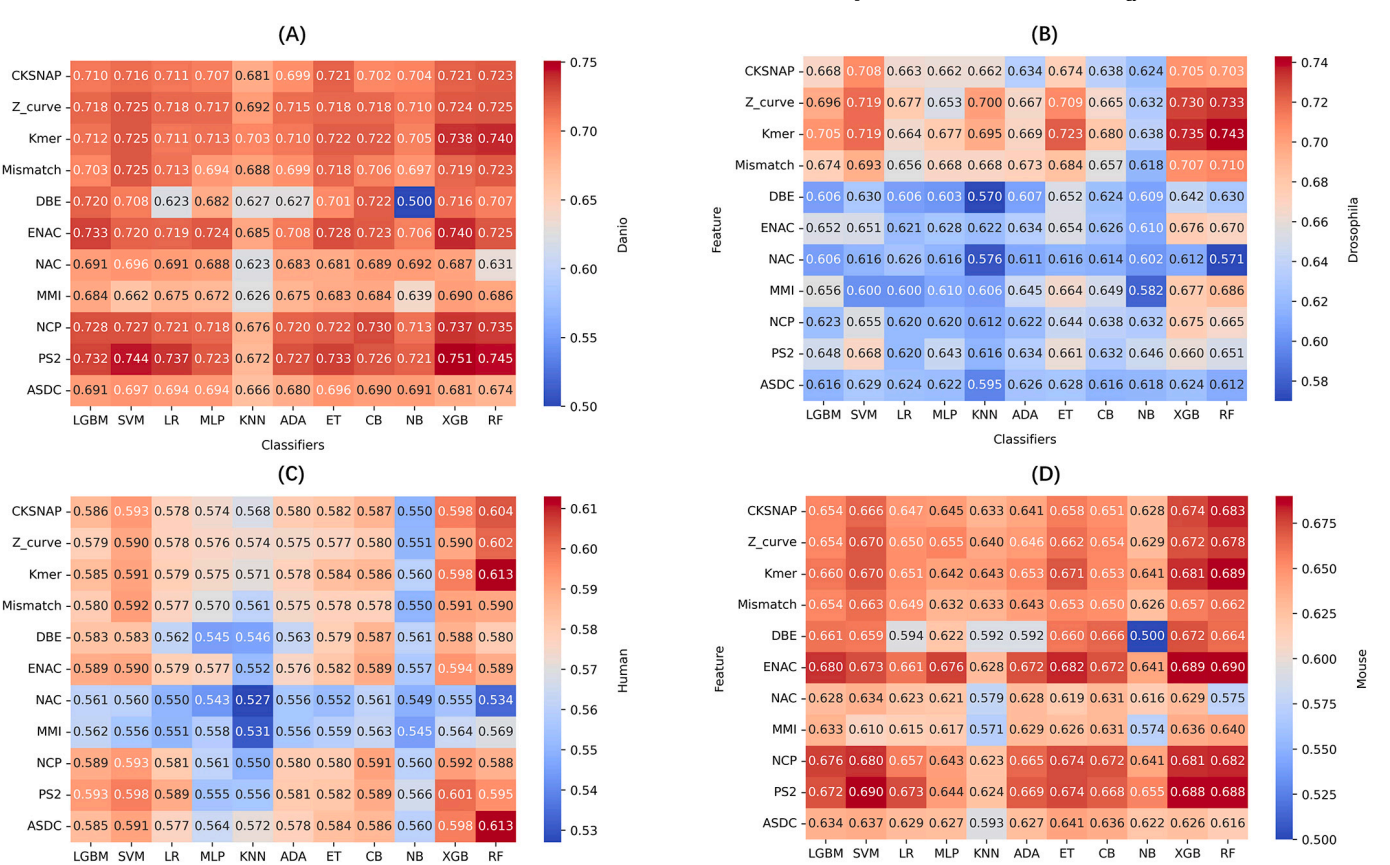
Performance comparison of different classifiers using various features across four species: (A) Danio, (B) Drosophila, (C) Human, (D) Mouse. Each heatmap displays the accuracy of 11 feature encodings combined with different ML classifiers, showing their impact on model performance. The color scale reflects the accuracy, with darker shades indicating better results.

The heatmaps in Fig. 2 display the accuracy of various feature sets combined with different classifiers for m5C site prediction across four species: danio, drosophila, human, and mouse. The color intensity in each heatmap reflects the classification accuracy, with darker shades indicating better performance.

For danio, CKSNAP consistently yields high accuracy values, particularly when paired with classifiers like XGBoost and RF. These features demonstrate strong predictive power, highlighting their effectiveness in capturing the relevant sequence patterns for danio. Similarly, in drosophila, Z_curve and Kmer are the most effective features, with XGBoost and RF again delivering the best results, demonstrating their capacity to handle the complexities of this species' data. In the human dataset, the performance is relatively lower compared to the other datasets, with Kmer paired with RF providing the highest accuracy (0.613). The mouse dataset, on the other hand, shows consistent high performance, with PS2 and ENAC features producing the best results, especially when combined with XGBoost and RF. These feature-classifier combinations lead to high accuracy values, reflecting the strength of these models in predicting m5C sites in mouse RNA.

Across all datasets, ensemble classifiers such as XGBoost and RF consistently outperform simpler models like KNN and NB. This emphasizes the importance of using more complex classifiers to capture intricate relationships between features and m5C sites, while simpler methods tend to struggle with the complexity of RNA sequences and m5C modifications.

Furthermore, to demonstrate the statistical significance of the stacked model over the baseline model, we performed a rigorous statistical analysis on the mouse independent dataset using the absolute prediction error as an assessment metric [45], [46]:

(6) Absolute Prediction Error=|predicted probability−true label|

Both paired t-tests and Wilcoxon signed-rank tests were applied to assess the performance differences [47], [48]. As shown in Table S5, on the independent dataset, the stacking model achieved statistically significant improvements compared to almost all base models (p<0.001 for both tests), confirming its superiority. Although the differences with some models (e.g., GaussianNB_cksnap) are small, the stacking model still demonstrated statistically significant enhancements (p = 0.013 for the t-test and p = 0.001 for the Wilcoxon test), highlighting the value of the stacking approach in improving predictive accuracy.

In conclusion, our comprehensive analysis demonstrates two key findings for optimal m5C site prediction. Firstly, the heatmaps clearly demonstrate the importance of selecting the right feature sets and classifiers for optimal m5C site prediction. Features such as CKSNAP and Kmer stand out as effective across multiple species, while ensemble classifiers like XGBoost and RF deliver superior performance. Furthermore, we provide rigorous statistical validation of the stacked model's advantages. Through paired t-tests and Wilcoxon signed-rank tests (p<0.001 against most base models), our results confirm that the stacking approach achieves statistically significant improvements in prediction accuracy. Together, these results not only guide feature and classifier selection for future studies but also quantitatively validate the stacking strategy's ability to synthesize the strengths of individual models, establishing a robust framework for RNA m5C site prediction.

### 3.4 Effect of the ensemble strategy on prediction performance

Recent studies have demonstrated the effectiveness of ensemble methods in bioinformatics tasks. For instance, Abbas et al. proposed an ensemble framework using a multi-model voting approach for anticancer peptide classification [49]. Inspired by the success of Voting in such tasks, we further explored the potential of ensemble strategies by comparing the performance of Stacking and Voting methods for m5C site prediction.

**Fig. 3:**
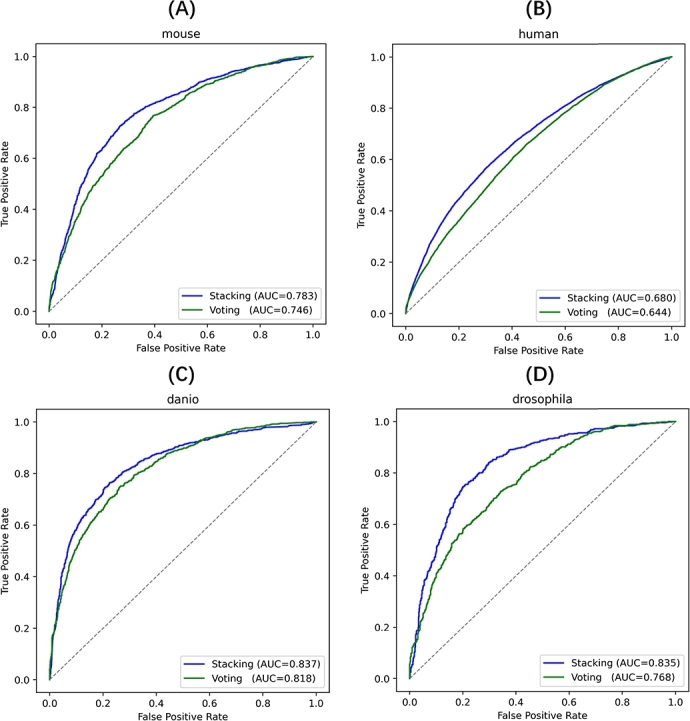
ROC curves for the independent dataset comparing the performance of Stacking and Voting methods across four species: (A) Danio, (B) Drosophila, (C) Human, (D) Mouse. The curves demonstrate that Stacking consistently outperforms Voting in terms of AUC across all species, confirming the superiority of the Stacking method for RNA modification site prediction.

To evaluate the relative effectiveness of these two methods, the ROC curves in Fig. 3 compare the performance of Stacking and Voting methods on the independent dataset across four species. The results consistently show that Stacking outperforms Voting in all species in terms of AUC values, which reflects a superior model performance for predicting m5C sites.

In the case of mouse, Stacking method consistently achieves higher AUC compared to Voting method, indicating better differentiation between m5C and non-m5C. A similar trend is observed across other species, with Stacking demonstrating an enhanced ability to capture complex data patterns and deliver more robust results. While both methods perform well, Stacking method provides better accuracy, particularly in datasets like danio and mouse, where the improvement is more pronounced. These findings highlight Stacking method's effectiveness in distinguishing subtle differences in RNA modifications, making it a more reliable approach for RNA modification profiling.

### 3.5 Performance analysis of different meta-classifiers

**Fig. 4:**
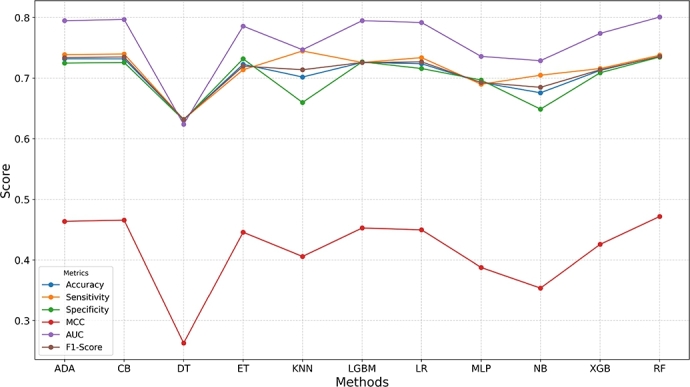
Performance analysis using different meta classifiers on the drosophila dataset.

The performance analysis, as shown in Fig. 4 and Table S2, compares the effectiveness of various meta-classifiers on the drosophila dataset: AdaBoost (ADA), CatBoost (CB), Decision Tree (DT), Extra Trees (ET), K-Nearest Neighbors (KNN), LightGBM (LGBM), Logistic Regression (LR), Multi-Layer Perceptron (MLP), Naive Bayes (NB), XGBoost (XGB), and Random Forest (RF) across multiple evaluation metrics.

From the analysis, it is clear that RF outperforms all other classifiers across most metrics. The performance of RF is consistently strong, with an accuracy of 0.736, Sn of 0.738, Sp of 0.735, MCC of 0.472, AUC of 0.801, and F1 score of 0.736, indicating that RF is the most effective classifier for m5C site prediction in this case.

ADA and CB follow closely behind, both achieving an accuracy of 0.732, with very similar performance across all metrics. These classifiers, along with LGBM and LR, perform consistently well, with accuracy values ranging from 0.724 to 0.732, and strong results in AUC and F1 score, demonstrating their suitability for this task. On the other hand, classifiers like DT and NB show relatively lower performance, particularly in metrics such as MCC, AUC, and F1 score. DT has an accuracy of 0.632, with a MCC of 0.263, indicating that it struggles to classify m5C sites effectively. Similarly, NB performs the worst in terms of accuracy (0.676) and MCC (0.354), suggesting it is less suited for this particular problem.

Finally, KNN and XGB also perform decently, with XGB showing slightly higher results compared to KNN in terms of sensitivity and specificity, but falling short in comparison to RF and CB.

In summary, RF stands out as the best meta-classifier for m5C site prediction, offering the highest performance across all metrics. While other classifiers such as ADA, CB, and LGBM also perform well, simpler classifiers like DT and NB show limitations. This analysis demonstrates the importance of choosing the right classifier, RF offering superior performance for this task.

### 3.6 Visualization of m5C site prediction patterns

t-SNE (t-Distributed Stochastic Neighbor Embedding) [40], a widely employed nonlinear dimensionality reduction method, specializes in projecting high-dimensional data into intuitive 2D or 3D visualizations. By focusing on preserving local neighborhood relationships between data points, it clusters similar samples together and separates dissimilar ones, which makes it particularly effective for revealing hidden patterns in complex datasets. In machine learning workflows, t-SNE is often used to evaluate how effectively a model captures the underlying data representation.

In this study, we input the output probabilities of each classifier into the t-SNE algorithm. These probabilities represent the likelihood that each sample will be categorized as either m5C or non-m5C based on the predictions of the classifier. By applying t-SNE to these output probabilities, we can observe the model's ability to distinguish between m5C and non-m5C sites on different datasets.

**Fig. 5:**
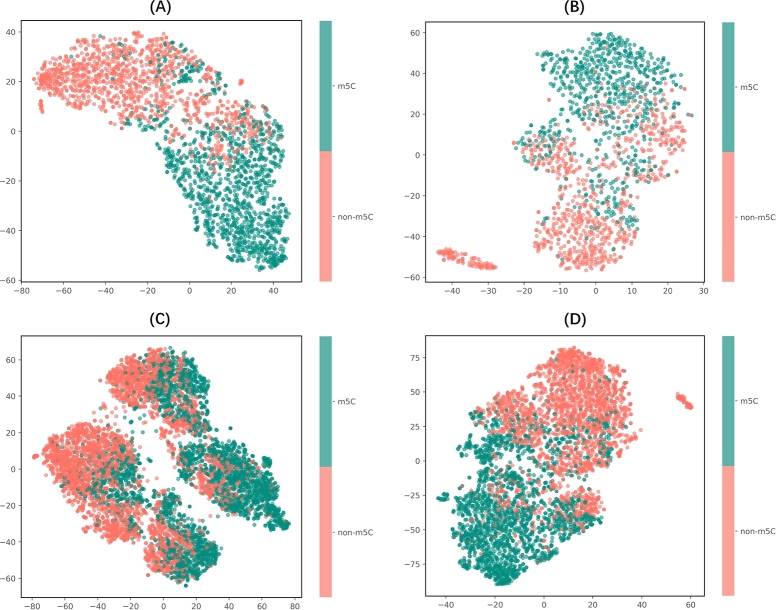
t-SNE visualization of m5C and non-m5C sites for four species: (A) Danio, (B) Drosophila, (C) Human, (D) Mouse. The scatter plots show the distribution of m5C-modified (green) and non-modified (red) sites, with the t-SNE algorithm effectively capturing the separation between the two classes across the different species.

The t-SNE plots in Fig. 5 show the distribution of m5C (green) and non-m5C (red) sites across four species. For the danio and drosophila datasets, the m5C and non-m5C sites are clearly separated, indicating that the model has effectively captured the distinguishing features of m5C modifications in these species. Similarly, the mouse dataset shows even stronger clustering, with effective separation between the two classes, suggesting that the model performs exceptionally well for this species.

For the human dataset, the t-SNE plot reveals more overlap between the m5C and non-m5C sites compared to other species. While a clear clustering is still visible, the separation between the two classes is less distinct, indicating that the model faces some challenges in distinguishing m5C sites in human RNA. This could be due to the more complex nature of m5C modifications in human RNA or limitations in the feature sets used. Nevertheless, the model still manages to group most of the m5C and non-m5C sites separately, and further improvements in the feature extraction process may improve the discrimination.

In conclusion, the t-SNE results in Fig. 5 highlight the strong performance of the model across species. The clear clustering of m5C and non-m5C sites in the dataset across species demonstrates the effectiveness of the model. This visualization approach provides valuable insight into how well the model can distinguish between m5C and non-m5C sites in different biological contexts.

### 3.7 Cross-species comparative performance analysis

**Fig. 6:**
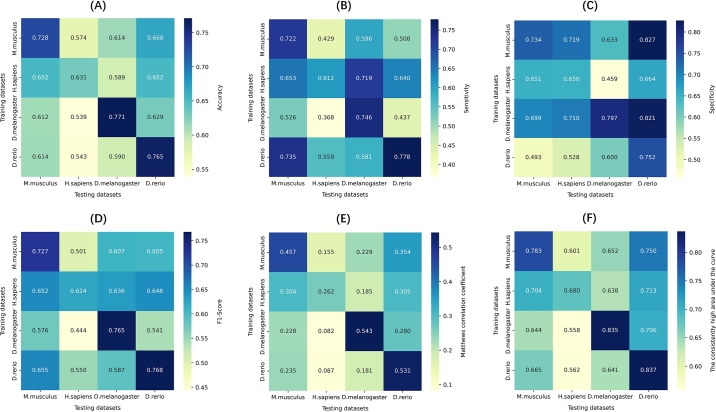
Cross-species analysis of the model using various metrics: (A) Accuracy, (B) Sensitivity, (C) Specificity, (D) F1-Score, (E) Matthews Correlation Coefficient (MCC), (F) The consistency high area under the curve.

The heatmaps in Fig. 6 provide a cross-species performance comparison of the model, evaluating its ability to predict m5C sites when trained and tested on different species. The cross-species performance analysis revealed significant variations in effectiveness depending on the training and testing species combinations.

For mouse, the model performed well when tested on human, achieving relatively high accuracy and sensitivity. However, performance decreased when tested on drosophila, suggesting challenges in transferring learned patterns from mammals to insects.

Human datasets showed moderate results, with decent performance when tested on mouse, but poorer results when tested on danio and drosophila. This reflects the difficulties of applying human-based datasets to more distant species. The challenges in predicting human RNA modifications may stem from the complexity of human RNA biology, including the presence of more diverse RNA-binding proteins, intricate regulatory networks, and potentially unique methylation patterns. Further investigation into these biological factors, such as the role of specific RNA-binding proteins or the influence of tissue-specific expression patterns, could help explain the observed performance gaps and improve the model's applicability to human data.

Drosophila posed significant challenges when the dataset was tested on other species, particularly human and danio, resulting in lower accuracy and F1-scores. This suggests that the unique evolutionary adaptations in insect RNA modifications, which differ significantly from those in vertebrates, may hinder the transferability of models trained on vertebrate data. The distinct RNA modification landscape in drosophila, including differences in methylation enzymes and regulatory mechanisms, likely contributes to these performance limitations.

Notably, danio provided the most consistent results across all species, with high accuracy, sensitivity, and specificity. This indicates that testing on the danio dataset resulted in robust model performance across diverse species, possibly due to the evolutionary conservation of certain RNA modification pathways in zebrafish that are shared with other vertebrates.

Overall, these results suggest that while the model is generally applicable across species, performance differences indicate that species-specific adjustments may be necessary to enhance accuracy and reliability. The model's ability to maintain strong performance with certain species, like danio, highlights its potential for broad applicability with further refinement. Further research into the biological factors underlying these performance differences, particularly in human RNA and the unique challenges posed by drosophila, could lead to more effective cross-species predictions.

### 3.8 Model interpretability analysis

**Fig. 7:**
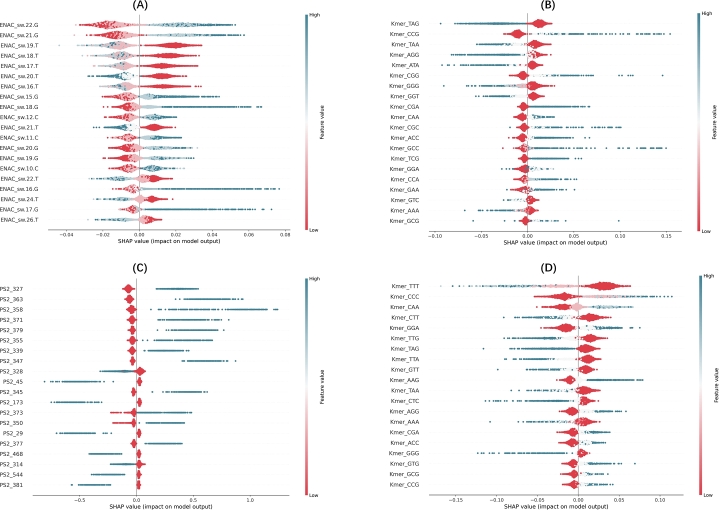
SHAP values for the top-performing model for each dataset: (A) Danio (ENAC_XGB), (B) Drosophila (Kmer_RF), (C) Mouse (PS2_SVM), (D) Human (Kmer_RF). These plots highlight the importance and impact of each feature on the model's predictions, with high SHAP values indicating features that contribute more significantly to predicting m5C-modified sites.

**Fig. 8:**
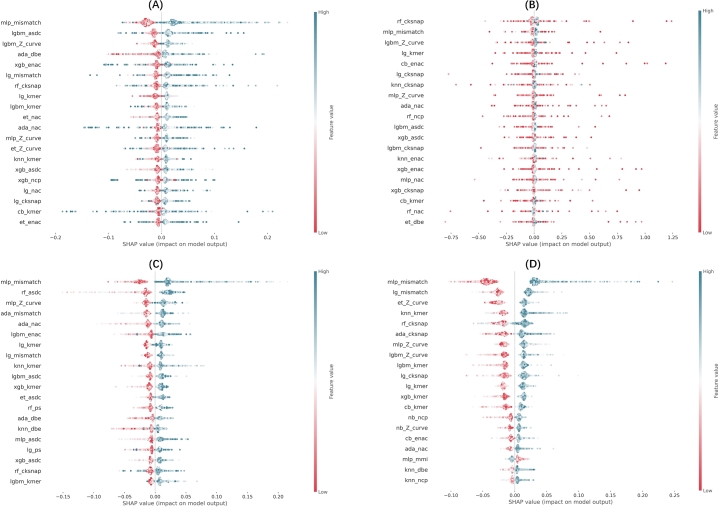
Meta-classifier's SHAP values: (A) Danio, (B) Drosophila, (C) Human, (D) Mouse.

The SHAP (Shapley Additive Explanations) values presented in Fig. 7, Fig. 8 provide a detailed analysis of feature importance for the top-performing model and the meta-classifier across four species [41]. These charts illustrate the impact of different features on the model's predictions and can help us understand the factors that drive the decision-making process.

In Fig. 7, which focuses on the top-performing model for each dataset, we see a strong variation in feature importance. For instance, in the mouse dataset, the ENAC_sw.22.G feature has a significant impact on model output. Similarly, in the drosophila dataset, Kmer_TAG exhibits substantial effects, with higher SHAP values. This suggests that these features are crucial in distinguishing m5C sites in these species. The danio dataset highlights the importance of features such as PS2_327, which influence model predictions more strongly compared to other features.

Fig. 8's SHAP value analysis shows that the meta-classifier incorporates diverse features from different models. For example, mlp_mismatch and rf_cksnap show a substantial impact on the model's predictions for the human dataset, as they occupy the highest positions in terms of SHAP values. This demonstrates that the meta-classifier can effectively integrate these features to improve prediction performance. Similarly, in drosophila, mlp_mismatch and lg_mismatch have a strong influence, underlining the flexibility of the meta-classifier in handling diverse input from various classifiers.

In summary, SHAP value analyses emphasize the significance of certain features in accurately predicting m5C sites and demonstrate how the meta-classifier improves model performance by integrating valuable features from various classifiers. This deeper insight into feature contributions strengthens both the model's interpretability and predictive power.

### 3.9 Web server interface

**Fig. 9:**
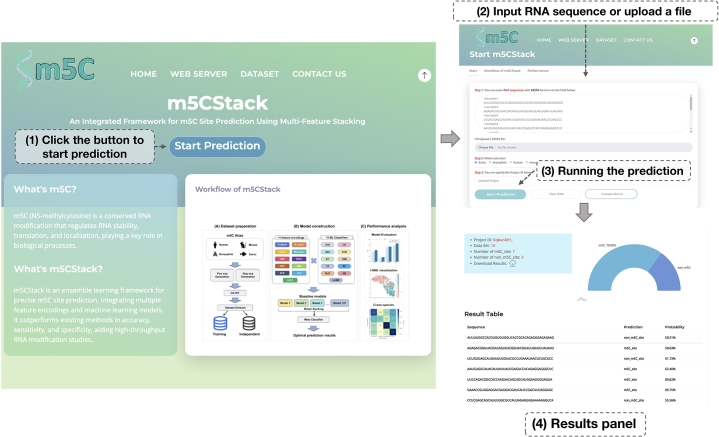
Demonstration of the m5CStack web interface. (1) Starting the prediction: Users initiate the process by clicking the Start Prediction button, which opens a dedicated page for sequence input. (2) Input RNA sequences: On the new page, users can input RNA sequences directly into the text box or upload a file containing multiple sequences by clicking the Upload button. (3) Running the prediction: After inputting the sequences, users click the Start Prediction button to submit the sequences for analysis. (4) Results panel: The results page displays a summary of the prediction outcomes.

For user convenience, m5CStack is implemented as a user-friendly web interface (Fig. 9), freely accessible at https://awi.cuhk.edu.cn/~biosequence/m5CStack/. Users can start by clicking the “Start Prediction” button, which opens a page to input RNA sequences directly or upload a file containing multiple sequences. Upon submission, the system processes the input and displays the predicted m5C modification sites, along with confidence scores from the model in an intuitive format.

## 4 Conclusion

This study introduces m5CStack, an advanced ensemble learning framework designed to predict RNA 5-methylcytosine (m5C) modification sites. m5CStack integrates a number of different feature coding techniques and classifiers into a stacked architecture that significantly improves prediction accuracy, sensitivity, and robustness compared to existing approaches. The diversity of these classifiers enables the model to capture complex sequence patterns, while the meta-classifiers effectively combine their outputs to improve inter-species reliability. In addition, extensive evaluation across multiple species further highlights the high performance and versatility of the model, while feature importance analysis using SHAP values provides valuable insights into the key contributions of specific sequence features, improving the interpretability and biological relevance of the model, thus making m5CStack a powerful and reliable tool for RNA modification profiling.

Despite these strengths, m5CStack does come with certain limitations. The large-scale dataset and the integration of multiple classifiers result in longer training times, which can be a challenge in high-throughput or time-sensitive applications. Furthermore, while the model excels at predicting m5C sites, its generalization to other RNA modifications remains uncertain, and further refinement is needed for broader applicability.

In conclusion, m5CStack represents a significant advancement in RNA modification research. Its ability to deliver high-accuracy predictions and provide interpretability makes it a powerful tool for exploring the role of m5C modifications in RNA biology, disease mechanisms, and epigenetic regulation. While the large-scale dataset and integration of multiple classifiers result in longer training times, this limitation can be mitigated through some practical strategies. For instance, employing more efficient ensemble learning techniques, such as early stopping or model distillation, can streamline computational demands while maintaining predictive accuracy. Additionally, future work will focus on improving the model's efficiency by exploring lightweight architectures, parallel computing, or distributed training frameworks, as well as extending its applicability to other RNA modifications. These enhancements will ensure that m5CStack remains a versatile and scalable tool for high-throughput applications and broader molecular biology studies.

## 5 Key points

• m5CStack is a novel ensemble learning framework designed to predict RNA 5-methylcytosine (m5C) modification sites with high accuracy.
• The model integrates multiple distinct feature encoding techniques and ML classifiers, using a stacking ensemble strategy to improve prediction robustness.
• Experimental results demonstrate that m5CStack achieves state-of-the-art performance, outperforming existing methods with enhanced accuracy and reliability across multiple species.
• SHAP-based feature significance analysis and cross-species validation improve the model's interpretability and generalizability.

## Funding

This work was supported by 10.13039/501100010877Shenzhen Science and Technology Innovation Commission (JCYJ20230807114206014), the Guangdong Province Basic and Applied Basic Research Fund (2021A1515012447), 10.13039/501100001809National Natural Science Foundation of China (32070659), The Kobilka Institute of Innovative Drug Discovery, 10.13039/100022813The Chinese University of Hong Kong, Shenzhen, China, and the Warshel Institute for Computational Biology funding from Shenzhen City and Longgang District (LGKCSDPT2024001).

## CRediT authorship contribution statement

**Xuxin He:** Writing – original draft, Validation, Methodology, Conceptualization. **Jiahui Guan:** Writing – original draft, Validation, Methodology, Conceptualization. **Peilin Xie:** Validation, Formal analysis. **Zhihao Zhao:** Validation, Formal analysis. **Qianchen Liu:** Writing – original draft. **Lantian Yao:** Project administration, Conceptualization. **Ying-Chih Chiang:** Supervision, Project administration, Funding acquisition, Conceptualization.

## Declaration of Competing Interest

The authors have declared no conflict of interest.

## Data Availability

The source code and data of this study can be found in a GitHub repository: https://github.com/okkk11111/m5CStack. Web server is available at https://awi.cuhk.edu.cn/~biosequence/m5CStack/.

## Appendix A Supplementary material

MMC Information about the details of feature encoding algorithms; Bar chart evaluation of model performance on the training and independent datasets; Summary of hyperparameters of different machine learning models; Performance evaluation using different meta classifiers; Performance comparison between our method and the simplified method; Training and testing time comparison between our method and the simplified method; and statistical evaluation of the m5CStack model compared to baseline models. MMC
