# Supplementary material for: m5CStack: An integrated framework for m5C site prediction using multi-feature stacking
Source: Comput Struct Biotechnol J. 2025 May 12;27:1901–12. doi: 10.1016/j.csbj.2025.05.004 (PMC12145772; doi:10.1016/j.csbj.2025.05.004)
Supplement: MMC — Information about the details of feature encoding algorithms; Bar chart evaluation of model performance on the training and independent datasets; Summary of hyperparameters of different machine learning models; Performance evaluation using different meta classifiers; Performance comparison between our method and the simplified method; Training and testing time comparison between our method and the simplified method; and statistical evaluation of the m5CStack model compared to baseline models. [file mmc1.pdf]

SUPPLEMENTARY MATERIALS

Supplementary Methods

Details of feature encoding algorithms:

1. CKSNAP (Composition of k-spaced nucleic acid pair)

The CKSNAP feature encoding calculates the frequency of nucleic acid pairs that are separated by any k nucleic acid (where k = 0, 1, 2, …, 5). For example, when k = 0, there are 16 possible 0-spaced nucleic acid pairs, such as 'AA', 'AC', 'AG', 'AU', 'CA', 'CC', 'CG', 'CU', 'GA', 'GC', 'GG', 'GU', 'UA', 'UC', 'UG', and 'UU'. A feature vector can be defined as follows:

$$\left(\frac{N_{AA}}{N_{total}}, \frac{N_{Ac}}{N_{total}}, \frac{N_{AG}}{N_{total}}, \ldots, \frac{N_{UU}}{N_{total}}, \right)_{16}.$$

Each descriptor value represents the composition of the corresponding nucleic acid pair in the nucleotide sequence. For instance, if the nucleic acid pair 'AA' appears m times in the sequence, its composition is the ratio of m to the total number of 0-spaced nucleic acid pairs (Ntotal) in the sequence. For different values of k (0, 1, 2, 3, 4, 5), the value of Ntotal is P – 1, P – 2, P – 3, P – 4, P – 5, and P – 6, respectively, where P is the length of the nucleotide sequence. Gap value is the length of k-spaced nucleic acid, default is 5.

2. Z_curve (The Z curve parameters for frequencies of phase-independent tri-nucleotides)

The Z_curve descriptor consider the frequency of tri-nucleotides, denoted by p(XY), where X, Y = A, C, G and U. This descriptor can be calculated as follows:

$$\begin{cases} x_{XY} = (p(XYA) + p(XYG)) - (p(XYC) + p(XYU)), \\ y_{XY} = (p(XYA) + p(XYC)) - (p(XYG) + p(XYU)), \\ z_{XY} = (p(XYA) + p(XYU)) - (p(XYG) + p(XYC)), \\ \qquad X = A, C, G, U; Y = A, C, G, U \end{cases}$$

3. Kmer

For the kmer descriptor, RNA sequences are represented by the occurrence frequencies of k neighboring nucleic acids. This method has been effectively used in predicting human gene regulatory sequences and identifying enhancers. The calculation of the Kmer (k=3) descriptor is as follows:

$$f(t) = \frac{N(t)}{N}, \quad t \in \{AAA, AAC, AAG, \ldots, UUU\},$$

where N(t) represents the count of kmer type t, and N is the length of the nucleotide sequence. Kmer size is the number of neighboring nucleic acids, default is 3.

4. Mismatch (The occurrence of kmers, allowing at most m mismatches)

The mismatch profile calculates kmer occurrences with a maximum of m inexact matches (where m < k). This descriptor has two parameters: k, representing neighboring nucleic acids, and m, the number of inexact matches. It is defined as follows:

$$f_{k,m} = \left(\sum_{j=0}^{m} c_{1,j}, \sum_{j=0}^{m} c_{2,j}, \ldots, \sum_{j=0}^{m} c_{4^k,j}, \right),$$

Here, ci,j refers to the occurrences of the i-th kmer type with j mismatches, where i = 1, 2, 3, …, 4k, and j = 0, 1, 2, …, m. Kmer size is the number of k neighboring nucleic acids, default is 3. Mismatch value is the nucleotide number of inexact matching (< kmer size), default is 1.

5. DBE (Dinucleotide binary encoding)

The dinucleotide binary encoding descriptor captures the positional information of each dinucleotide within a sequence. There are 16 possible dinucleotides in total. In this descriptor, each dinucleotide is encoded into a 4-dimensional 0/1 vector. For example, AA is represented as (0,0,0,0); AU as (0,0,0,1); AC as (0,0,1,0); and so on, with GG encoded as (1,1,1,1). By using the dinucleotide binary encoding, we can generate a 160 (=40×4)-dimensional 0/1 vector for a given sequence.

6. NAC (Nucleic Acid Composition)

The Nucleic Acid Composition (NAC) encoding determines the frequency of each nucleic acid type within a nucleotide sequence. The frequencies for the 4 natural nucleic acids (i.e., "A, C, G, U") are calculated as follows:

$$f(t) = \frac{N(t)}{N}, \quad t \in \{A, C, G, U\},$$

where N(t) represents the count of nucleic acid type t, and N is the total length of the nucleotide sequence.

7. ENAC (Enhanced nucleic acid composition)

The Enhanced Nucleic Acid Composition (ENAC) calculates the NAC within a sequence window of fixed length (the default is 5), which slides continuously from the 5' to 3' terminus of each nucleotide sequence. This approach is typically used to encode a nucleotide sequence with equal length segments.

Sliding window size is the length of the sliding window, default is 5.

8. MMI (Multivariate mutual information)

To apply multivariate mutual information on a RNA sequence, the '2-mer' set T2 = {AA, AC, AG, AU, CC, CG, CU, GG, GU, UU} and '3-mer' set T3 = {AAA, AAC, AAG, AAU, ACC, ACG, ACU, AGG, AGU, AUU, CCC, CCG, CCU, CGG, CGU, CUU, GGG, GGU, GUU and UUU} are defined. The mutual information can then be calculated as follows:

$$I(N_1N_2) = f(N_1N_2)\ln\frac{f(N_1N_2)}{f(N_1)f(N_2)}$$

$$I(N_1N_2N_3) = f(N_1N_2)\ln\frac{f(N_1N_2)}{f(N_1)f(N_2)} + \frac{f(N_1N_3)}{f(N_3)}\ln\frac{f(N_1N_3)}{f(N_3)} - \frac{f(N_1N_2N_3)}{f(N_2N_3)}\ln\frac{f(N_1N_2N_3)}{f(N_2N_3)}$$

Here, f(Ni) represents the frequency of the i-th element in the sequence, f(Ni,Nj) refers to the frequency of the 2-mer element NiNj, and f(Ni,Nj,Nk) denotes the frequency of the T3's element NiNjNk.

9. NCP (Nucleotide chemical property)

RNA consists of four distinct nucleotides: adenine (A), guanine (G), cytosine (C), and uracil (U), each with unique chemical structures and binding properties. These nucleotides can be categorized into three groups based on their chemical characteristics:

Chemical property	Class	Nucleotides
Ring Structure	Purine	A, G
	Pyrimidine	C, U
Functional Group	Amino	A, C
	Keto	G, U
Hydrogen Bond	Strong	C, G
	Weak	A, U

Specifically, A is represented by the coordinates (1, 1, 1), C by (0, 1, 0), G by (1, 0, 0), and U by (0, 0, 1).

10. PS2 (Position-specific of two nucleotides)

There are 16 possible pairs of adjacent nucleotides (4 × 4 = 16), such as AA, AU, AG, and so on. Each pair is one-hot (i.e. binary) encoded into 16 binary variables. For example, AA is encoded as (1000000000000000), AC as (0100000000000000), and so forth. The sequence AAC would be encoded as (10000000000000000100000000000000).

11. ASDC (Adaptive skip dinucleotide composition)

The adaptive skip dipeptide composition (ASDC) is a modified version of the dinucleotide composition that takes into account the correlation information not only between adjacent residues but also between intervening residues. For given a sequence, the feature vector for ASDC is represented by:

$$\mathrm{ASDC} = (\mathrm{f_{v1}}, \mathrm{f_{v1}}, \dots, \mathrm{f_{v16}}),$$

where f_{vi} is calculated by

$$\mathrm{f_{vi}} = \frac{\sum_{g=1}^{L-1} O_i^g}{\sum_{i=1}^{16} \sum_{g=1}^{L-1} O_i^g},$$

where f_{vi} denotes the occurrence frequency of all possible dinucleotide with ≤ L-1 intervening nucleotides.

Supplementary Figures

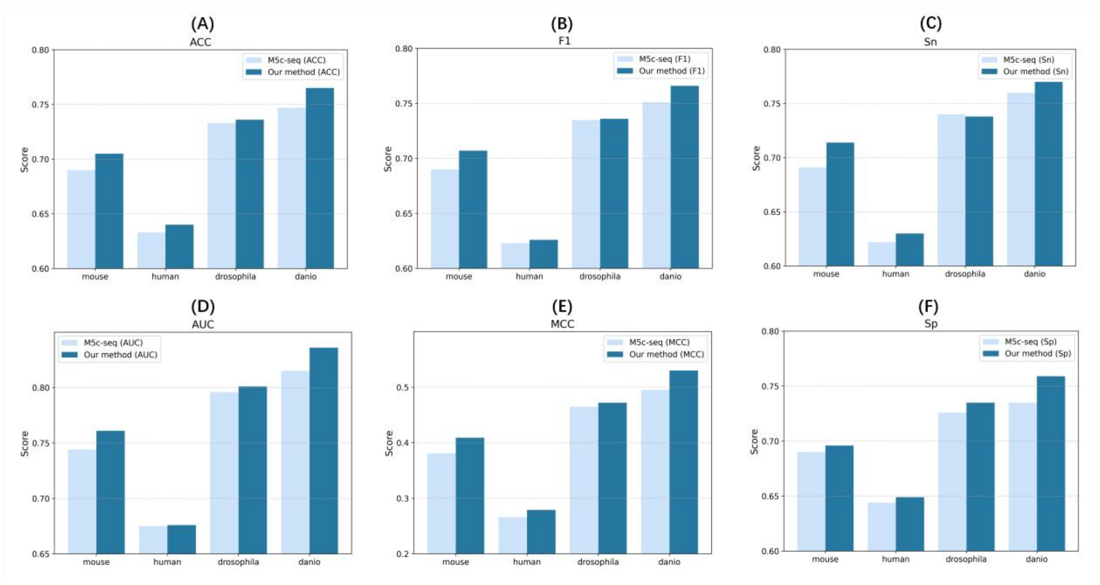

Figure S1. Bar chart evaluation of model performance using 5-fold cross-validation on the training dataset.

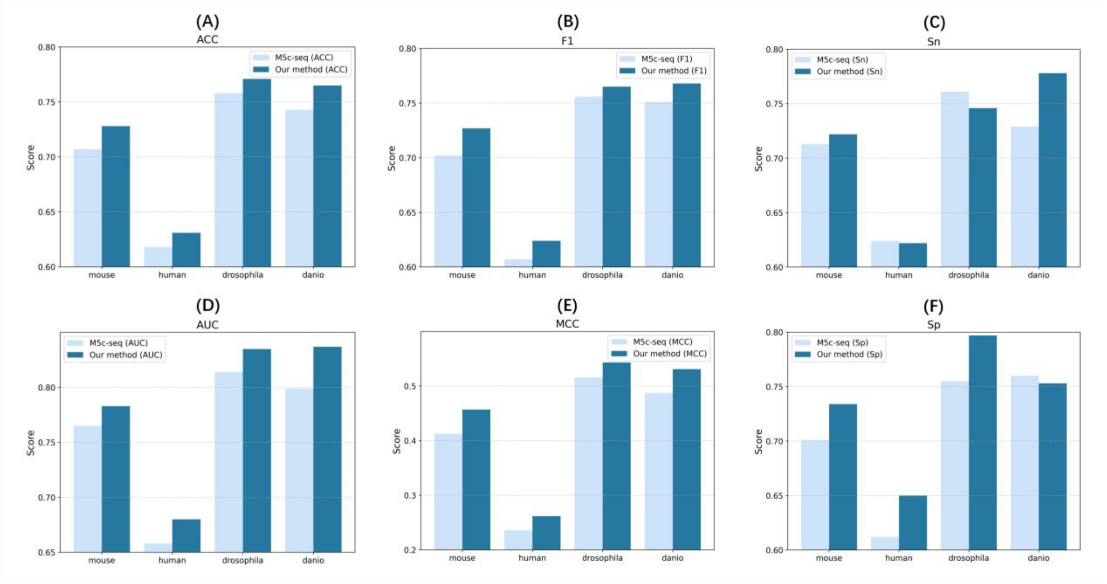

Figure S2. Bar chart evaluation of model performance on the independent dataset.

Supplementary Tables

Table S1. Summary of hyperparameters of different machine learning models.

Method	Parameter	Value
XGBClassifier	learning_rate	0.018
	max_depth	11
	min_child_weight	1
	gamma	0.174
	colsample_bytree	0.192
	n_estimators	625
	seed	14
LGBMClassifier	learning_rate	0.011
	max_depth	12
	min_child_samples	5
	min_child_weight	0.432
	min_split_gain	0.025
	n_estimators	471
	num_leaves	31
SVC	C	2
	kernel	rbf
	gamma	scale
RandomForestClassifier	max_depth	80
	bootstrap	False
	max_features	sqrt
	n_estimators	400
	min_samples_split	3
	random_state	0
LogisticRegression	penalty	l2
	C	1.0
	max_iter	1000
	solver	lbfgs
	random_state	14
MLPClassifier	hidden_layer_sizes	(210,)
	activation	logistic
	solver	adam
	random_state	10
GaussianNB	-	-
KNeighborsClassifier	n_neighbors	7
	weights	distance
	p	2
AdaBoostClassifier	base_estimator	DecisionTreeClassifier(max_depth=1)
	n_estimators	100

	learning_rate	0.5
	random_state	0
ExtraTreesClassifier	n_estimators	200
	max_depth	15
	min_samples_split	4
	min_samples_leaf	2
	max_features	sqrt
	bootstrap	True
	random_state	0
CatBoostClassifier	learning_rate	0.257
	max_depth	4
	iterations	90
	boosting_type	Plain

Table S2. Performance evaluation on drosophila training dataset using different meta classifiers.

Methods	ACC	Sn	Sp	MCC	AUC	F1
AdaBoost	0.732	0.739	0.725	0.464	0.795	0.734
CatBoost	0.732	0.740	0.726	0.466	0.797	0.735
DecisionTree	0.632	0.632	0.631	0.263	0.624	0.632
ExtraTrees	0.723	0.714	0.732	0.446	0.786	0.720
K-Nearest Neighbors	0.702	0.745	0.660	0.406	0.747	0.714
LightGBM	0.726	0.726	0.727	0.453	0.795	0.726
LogisticRegression	0.724	0.734	0.716	0.450	0.792	0.727
Multi-Layer Perceptron	0.693	0.690	0.697	0.388	0.736	0.693
Naive Bayes	0.676	0.705	0.649	0.354	0.729	0.685
XGBoost	0.713	0.716	0.709	0.426	0.774	0.714
Random Forest	0.736	0.738	0.735	0.472	0.801	0.736

Table S3. Performance comparison between our method and the simplified method.

Model	Accuracy	Sensitivity	Specificity	F1-score	MCC	AUC
Simplified method	0.693	0.689	0.697	0.692	0.386	0.732
Our method	0.728	0.722	0.734	0.727	0.457	0.783

Table S4. Training and testing time comparison between our method and the simplified method.

Model	Training time (seconds)	Testing time (seconds)
Simplified method	42.590	4.580
Our method	90.728	10.702

Table S5. Statistical evaluation of the m5CStack model compared to baseline models.

Model	p-value (t-test)	p-value (Wilcoxon)
LGBMClassifier_cksnap	<0.001	<0.001
LogisticRegression_cksnap	<0.001	<0.001
MLPClassifier_cksnap	<0.001	<0.001
KNeighborsClassifier_cksnap	<0.001	<0.001
AdaBoostClassifier_cksnap	<0.001	<0.001
ExtraTreesClassifier_cksnap	<0.001	<0.001
CatBoostClassifier_cksnap	<0.001	<0.001
GaussianNB_cksnap	0.013	0.001
XGBClassifier_cksnap	<0.001	<0.001
RandomForestClassifier_cksnap	<0.001	<0.001
LGBMClassifier_Z_curve	<0.001	<0.001
LogisticRegression_Z_curve	<0.001	<0.001
MLPClassifier_Z_curve	<0.001	<0.001
KNeighborsClassifier_Z_curve	<0.001	<0.001
AdaBoostClassifier_Z_curve	<0.001	<0.001
ExtraTreesClassifier_Z_curve	<0.001	<0.001
CatBoostClassifier_Z_curve	<0.001	<0.001
GaussianNB_Z_curve	0.011	<0.001
XGBClassifier_Z_curve	<0.001	<0.001
RandomForestClassifier_Z_curve	<0.001	<0.001
LGBMClassifier_kmer	<0.001	<0.001
LogisticRegression_kmer	<0.001	<0.001
MLPClassifier_kmer	<0.001	<0.001
KNeighborsClassifier_kmer	<0.001	<0.001
AdaBoostClassifier_kmer	<0.001	<0.001
ExtraTreesClassifier_kmer	<0.001	<0.001
CatBoostClassifier_kmer	<0.001	<0.001
GaussianNB_kmer	<0.001	<0.001
XGBClassifier_kmer	<0.001	<0.001
RandomForestClassifier_kmer	<0.001	<0.001
LGBMClassifier_mismatch	<0.001	<0.001
LogisticRegression_mismatch	<0.001	<0.001
MLPClassifier_mismatch	<0.001	<0.001
KNeighborsClassifier_mismatch	<0.001	<0.001
AdaBoostClassifier_mismatch	<0.001	<0.001
ExtraTreesClassifier_mismatch	<0.001	<0.001
CatBoostClassifier_mismatch	<0.001	<0.001
GaussianNB_mismatch	0.012	0.002
XGBClassifier_mismatch	<0.001	<0.001
RandomForestClassifier_mismatch	<0.001	<0.001
LGBMClassifier_dbe	<0.001	<0.001
LogisticRegression_dbe	<0.001	<0.001

MLPClassifier_dbe	0.058	0.091
KNeighborsClassifier_dbe	<0.001	<0.001
AdaBoostClassifier_dbe	<0.001	<0.001
ExtraTreesClassifier_dbe	<0.001	<0.001
CatBoostClassifier_dbe	<0.001	<0.001
GaussianNB_dbe	<0.001	<0.001
XGBClassifier_dbe	<0.001	<0.001
RandomForestClassifier_dbe	<0.001	<0.001
LGBMClassifier_enac	<0.001	<0.001
LogisticRegression_enac	<0.001	<0.001
MLPClassifier_enac	<0.001	<0.001
KNeighborsClassifier_enac	<0.001	<0.001
AdaBoostClassifier_enac	<0.001	<0.001
ExtraTreesClassifier_enac	<0.001	<0.001
CatBoostClassifier_enac	<0.001	<0.001
GaussianNB_enac	<0.001	<0.001
XGBClassifier_enac	0.032	0.022
RandomForestClassifier_enac	<0.001	<0.001
LGBMClassifier_nac	<0.001	<0.001
LogisticRegression_nac	<0.001	<0.001
MLPClassifier_nac	<0.001	<0.001
KNeighborsClassifier_nac	<0.001	<0.001
AdaBoostClassifier_nac	<0.001	<0.001
ExtraTreesClassifier_nac	<0.001	<0.001
CatBoostClassifier_nac	<0.001	<0.001
GaussianNB_nac	<0.001	<0.001
XGBClassifier_nac	<0.001	<0.001
RandomForestClassifier_nac	<0.001	<0.001
LGBMClassifier_mmi	<0.001	<0.001
LogisticRegression_mmi	<0.001	<0.001
MLPClassifier_mmi	<0.001	<0.001
KNeighborsClassifier_mmi	<0.001	<0.001
AdaBoostClassifier_mmi	<0.001	<0.001
ExtraTreesClassifier_mmi	<0.001	<0.001
CatBoostClassifier_mmi	<0.001	<0.001
GaussianNB_mmi	<0.001	<0.001
XGBClassifier_mmi	<0.001	<0.001
RandomForestClassifier_mmi	<0.001	<0.001
LGBMClassifier_ncp	<0.001	<0.001
LogisticRegression_ncp	<0.001	<0.001
MLPClassifier_ncp	0.026	0.041
KNeighborsClassifier_ncp	<0.001	<0.001
AdaBoostClassifier_ncp	<0.001	<0.001
ExtraTreesClassifier_ncp	<0.001	<0.001

CatBoostClassifier_ncp	<0.001	<0.001
GaussianNB_ncp	0.057	0.094
XGBClassifier_ncp	<0.001	<0.001
RandomForestClassifier_ncp	<0.001	<0.001
LGBMClassifier_ps	<0.001	<0.001
LogisticRegression_ps	<0.001	<0.001
MLPClassifier_ps	<0.001	<0.001
KNeighborsClassifier_ps	<0.001	<0.001
AdaBoostClassifier_ps	<0.001	<0.001
ExtraTreesClassifier_ps	<0.001	<0.001
CatBoostClassifier_ps	<0.001	<0.001
GaussianNB_ps	<0.001	<0.001
XGBClassifier_ps	<0.001	<0.001
RandomForestClassifier_ps	<0.001	<0.001
LGBMClassifier_asdc	<0.001	<0.001
LogisticRegression_asdc	<0.001	<0.001
MLPClassifier_asdc	<0.001	<0.001
KNeighborsClassifier_asdc	<0.001	<0.001
AdaBoostClassifier_asdc	<0.001	<0.001
ExtraTreesClassifier_asdc	<0.001	<0.001
CatBoostClassifier_asdc	<0.001	<0.001
GaussianNB_asdc	0.189	0.056
XGBClassifier_asdc	<0.001	<0.001
RandomForestClassifier_asdc	<0.001	<0.001
